# Transformation of adenocarcinoma to squamous cell carcinoma as a source of EGFR-TKI resistance: A case report and literature review

**DOI:** 10.3389/fonc.2022.942084

**Published:** 2022-09-08

**Authors:** Yun-Zhu Xi, Li Xie, Xiao-Wu Tan, Sai-Li Zeng

**Affiliations:** Department of Pulmonary and Critical Care Medicine, The Second Affiliated Hospital, Hengyang Medical School, University of South China, Hengyang, China

**Keywords:** adenocarcinoma, squamous cell carcinoma, epidermal growth factor receptor, tyrosine kinase inhibitor, transformation

## Abstract

In general, non-small cell lung cancer patients with epidermal growth factor receptor (EGFR) mutations respond to tyrosine kinase inhibitors (TKIs). However, most patients experience resistance within 1-2 years after treatment. The histological explanation for the acquired resistance is that malignant transformation occurs during cancer treatment. To date, the transformation from adenocarcinoma to squamous cell carcinoma associated with EGFR-TKI use remains poorly reported. We report a case of stage IV lung adenocarcinoma with EGFR mutations that converted to squamous cell carcinoma due to long-term administration of EGFR-TKIs. This report strengthens histological evolution as a source of acquired drug resistance.

## Introduction

At present, lung cancer remains as the leading cause of mortality worldwide (1). In China, the annual prevalence of lung cancer is estimated at 87.65/100,000.00 (2). Furthermore, its incidence and mortality rates have exhibited an increasing trend during the past decades (3). Lung cancer has two broad histological subtypes: (1) small-cell lung cancer (SCLC), approximately 15% of cases; (2) non-small-cell lung cancer (NSCLC), approximately 85% of cases. NSCLC can be further classified as adenocarcinoma, squamous cell carcinoma, and large-cell carcinoma (4). For NSCLC, although the histological type has a geographical distribution and varies between countries, adenocarcinoma is more common than squamous cell carcinoma (5). It is known that NSCLC patients with epidermal growth factor receptor (EGFR) mutations benefit from EGFR-tyrosine kinase inhibitor (TKI) therapy (6). Unfortunately, acquired resistance would eventually develop in almost all patients after treatment. EGFR T790M mutation is the primary mechanism of “first- or second-generation” EGFR-TKIs, which accounts for approximately 50% of cases (7). However, in a significant number of patients, the accurate mechanism of resistance remains unclear (8), and more research needs to be conducted to elucidate this. To date, as a source of EGFR-TKI resistance, the transformation from adenocarcinoma to squamous cell carcinoma remains poorly reported. Thus, we present a case of stage IV lung adenocarcinoma with EGFR mutations that converted to squamous cell carcinoma due to long-term administration of EGFR-TKIs. This case report would add to the understanding of the mechanism of acquired resistance.

## Case report

**Figure 1:**
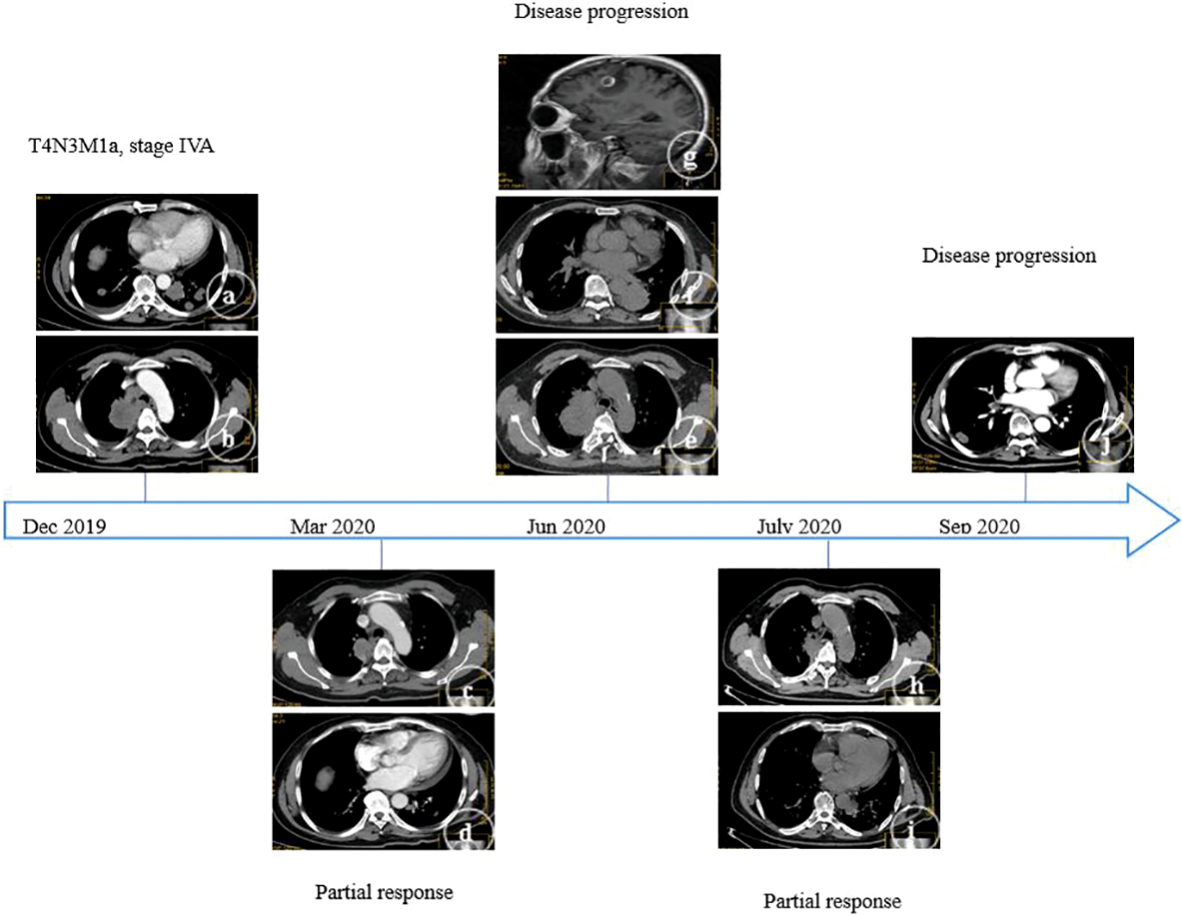
A series of CT findings during the clinical course. On admission, a primary lesion **(A)** in the right upper lobe of the lung and multiple metastases **(B)** in the left lower lobe of the lung were identified. Treatment with icotinib was performed, and after three months, partial remission was found (right, **C**; left, **D**). Treatment with icotinib was performed, and after six months, progressive disease was found (right, **E**; left, **F**; brain MRI, **G**). Treatment with osimertinib was performed, and after one month, partial remission was found (right, **H**; left, **I**). Treatment with osimertinib was performed, and after three months, progressive disease was found (right, **J**).

**Figure 2:**
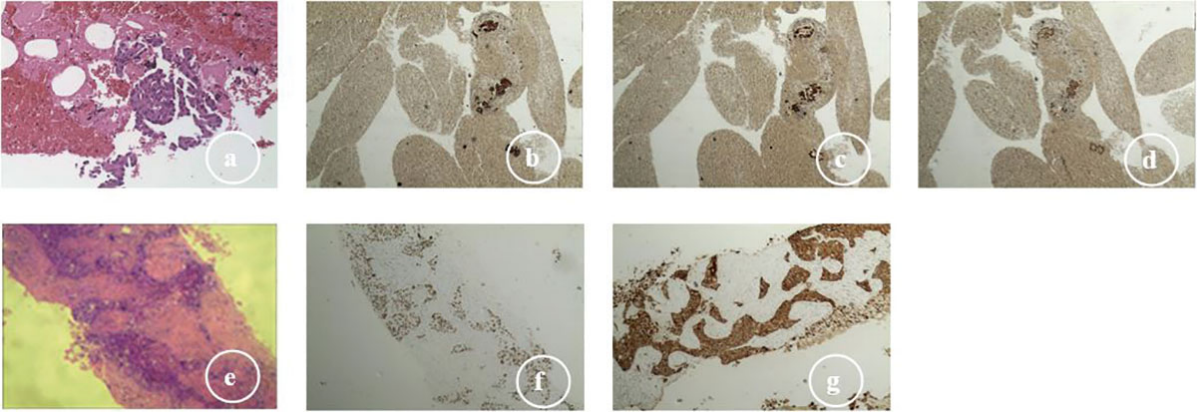
Histologic transformation from adenocarcinoma to squamous cell carcinoma: **(A)** lymph node specimens (EBUS-TBNA), adenocarcinoma (H&E, ×200); **(B)** adenocarcinoma CK7 (+); **(C)** adenocarcinoma TTF; (+) **(D)** adenocarcinoma NapsinA (+); **(E)** lung biopsies (CT-guided sampling), squamous cell carcinoma (H&E, ×400); **(F)** squamous cell carcinoma P40 (+); **(G)** squamous cell carcinoma CK5/6 (+).

In December 2019, a 59-year-old male (non-smoker) was admitted to our center due to cough for two weeks. The contrast-enhanced thoracic computed tomography (CT) revealed a primary lesion (lesion 1, 92×58 mm) in the right upper lobe of the lung and multiple metastases with a maximum size of 37×24 mm (lesion 2) in the left lower lobe of the lung, and distant spread to mediastinal lymph nodes was suspected (**Figures 1A, B**). Pathological investigations were performed on the lymph node specimens obtained by EBUS-TBNA, and the findings were, as follows: lung adenocarcinoma (**Figure 2A**) with TTF-1(+) (**Figure 2B**), NapsinA(+) (**Figure 2C**), CK7(+) (**Figure 2D**), D2-40(-), CD56(-), Wilms tumor(-), Caplonin(-), CDX-2(-), SATB2(-), Ki67(60% +), EGFR exon19 deletion and PD-L1 TPS 15%. Finally, the diagnosis of lung adenocarcinoma (T4N3M1a, stage IVA) with an Eastern Cooperative Oncology Group performance status (ECOG-PS) of 1 was made based on the clinical findings. Then, 125 mg of icotinib was orally administered, three times daily.

In March 2020, the thoracic CT revealed that the primary tumor and metastases decreased in size (**Figures 1C, D**): lesion 1 (42×39 mm) and lesion 2 (20×15 mm). Partial remission was also considered. However, in June 2020, the two lesions mentioned above enlarged (lesion 1, 100×80 mm; lesion 2, 65×62 mm), and a new lesion (lesion 3, 15×9 mm) was found in the right lower lobe (**Figures 1E, F**). Furthermore, multiple metastases were found in the left cerebellum and right frontal lobe by brain magnetic resonance imaging (MRI, **Figure 1G**). Therefore, the clinical response was evaluated as a progressive disease. The treatment was changed to pemetrexed (940 mg/day) and carboplatin (600 mg/day). The plasma next-generation sequencing (NGS) test revealed an EGFR T790M mutation, and that the EGFR exon 19 deletion did not disappear. Then, 80 mg of osimertinib was orally administered, once daily.

In July 2020, lesion 1 (69×41 mm, **Figures 1H, I**), lesion 2 (30×18 mm), and lesion 3 (10×6 mm) all decreased in size. Furthermore, an improvement was revealed by the brain MRI. The response was considered as partial remission. However, in August 2020, lesion 3 increased in size (25×14 mm, **Figure 1J**), and the pathological evidence of lung biopsies obtained by CT-guided procedures confirmed the squamous cell carcinoma (**Figure 2E**) with P40(+) (**Figure 2F**), CK5/6(+) (**Figure 2G**), CK7(-), NapsinA(-), TTF-1(-), CgA(-), CD56(-), Syn(-), Ki67(30% +), EGFR exon19 deletion, T790M mutant, MET amplification (high-throughput sequencing), and PD-L1 TPS 20%.

After the multi-disciplinary team (MDT) discussion, pembrolizumab (200 mg/day), paclitaxel liposome (250 mg/day) and carboplatin (600 mg/day) were incorporated into the present treatment with osimertinib. After two cycles of chemotherapy, in October 2020, a stable condition was suggested by the thoracic CT (lesion 1, 70×42 mm; lesion 2, 32×21 mm; lesion 3, 10×5 mm) and brain MRI (no obvious changes). Subsequently, the patient refused further chemotherapy, and only pembrolizumab was given for the third cycle of therapy. After one week, due to suspicion of cardiac injury, the use of pembrolizumab was interrupted, and osimertinib and crizotinib (250 mg, three times daily) were administered after the MDT meeting. The thoracic CT findings revealed that the patient’s condition improved again: lesion 1 (65×40 mm), lesion 2 (26×17 mm), and lesion 3 (disappeared). However, transient strabismus and unconsciousness suddenly occurred, and a lesion in the brain developed, as revealed by the MRI, which was treated with low-dose radiotherapy at 24 Gy in eight fractions.

In January 2021, the thoracic CT findings (lesion 1, 73×54 mm; lesion 2, 33×26 mm) revealed that the patient’s condition developed. This situation was further confirmed in May 2021 by CT imaging, and the ECOG-PS score was evaluated as 2. The treatment was switched to pembrolizumab (200 mg/day) and anlotinib (8 mg, three times daily, every three weeks, with one week intervals). In June 2021, the disease developed with enlarged lesions (lesion 1, 57×78 mm; lesion 2, 60×84 mm). Progressive status was considered, and the ECOG-PS score was evaluated as 3. Then, treatment with everolimus was initiated (5 mg, three times daily). Finally, the patient died in August 2021.

## Discussion

The mechanism of EGFR-TKI resistance can be classified as primary resistance and acquired resistance. Primary resistance is defined as the failure to respond to initial EGFR-targeted therapies, which accounts for approximately 60% of resistant events. Acquired resistance is defined by the following criteria: (1) treated with EGFR-TKI alone; (2) the EGFR mutants indicated for EGFR-TKIs and positive response (complete remission, partial remission, or stable disease after >6 months) to EGFR-TKIs; (3) progressive disease after 30 days of treatment with EGFR-TKIs. The factors associated with acquired resistance include T790M mutations, MET amplification, small cell transformation, and so on (9). In the present study, partial remission was achieved after the administration of icotinib. After six months, progressive disease was observed, and the T790M mutation, MET amplification, and squamous cell transformation were all confirmed in the present case. Furthermore, these three mechanisms are rarely reported in the same case.

In order to characterize the clinical characteristics of acquired resistance associated with the transformation from lung adenocarcinoma to squamous cell carcinoma, the investigators reviewed the case reports on this transformation. The PubMed database was searched for all relevant literature, and a total of 16 cases were collected (**Table 1**). Among these cases, 11 cases were female, and nine cases had a smoking habit. The mean period of transformation to squamous cell carcinoma was 12.2 ± 5.7 months, and the mean survival period after transformation was 7.1 ± 5.2 months. In addition, most of the initial mutants were drug-sensitive, and after the transformation, these patients still carried the mutants. These findings are consistent with the report of Roca et al. (24).

**Table 1:** Clinical characteristics of cases that converted from lung adenocarcinoma to squamous cell carcinoma.

References	Gender	Age (y)	Smoking	Initial EGFR mutations	Initial TKI	Remission period (months)	New EGFR mutations and others	Final regimes	Survival time (months)
Kuiper etal. (10)	F	63	N	ex21(L858R)	Gefitinib	5	PIK3CA	Gemcitabine, cisplatin, and erlotinib	6
Levin etal. (11)	F	66	N	ex19	Erlotinib	8	None	None	0
Jukna etal. (12)	F	79	N	ex19(del)	Erlotinib	15	T790M	Radiotherapy and gefitinib	7
Jukna etal. (12)	F	74	Y	ex21(L858R)	Gefitinib	10	T790M	Radiotherapy, vinorelbine, and carboplatin	11*
Okabe etal. (13)	M	69	Y	ex19(del)	Erlotinib	12	T790M	Osimertinib	3*
Hsieh etal. (14)	F	51	N	ex19(del)	Gefitinib	4	None	Surgery, gemcitabine, and cisplatin	6*
Hsieh etal., (14)	F	61	N	ex21(L858R)	Gefitinib	12	None	Erlotinib	0
Ding etal., (15)	F	56	N	ex19	Gefitinib	15	T790M、MET	Gemcitabine and osimertinib	17*
Yamaguchi etal. (16)	M	73	Y	ex19(del)	Afatinib	10	T790M	Osimertinib	12*
Uruga etal. (17)	M	61	Y	ex19(ins)	Erlotinib	28	T790M、PTEN	Osimertinib and pembrolizumab	17
Uruga etal. (17)	M	72	Y	ex21(L858R)	Erlotinib	9	T790M、PTEN	Osimertinib and pembrolizumab	8
Sato etal. (18)	F	52	Y	ex19(del)	Erlotinib	12	None	Afatinib	12*
Kong etal. (19)	F	64	N	ex21(L858R)	Afatinib	9	T790M	Osimertinib	7*
Longo etal. (20)	F	43	N	ex21(L858R)	Gefitinib	8	S768I	Gefitinib	3
Chiang etal. (21)	F	54	N	ex21(H835L,L833V),T790M	Gefitinib,Erlotinib,Afatinib and Osimertinib	19	TP53、mTOR	Osimertinib, paclitaxel, pembrolizumab, and everolimus	3*
Bruno etal., (22)	F	44	Y	ex19(del)	Afatinib	16	T790M	Osimertinib	2*
Bruno et al. (23)	M	38	Y	ex21(L858R)	Erlotinib	15	T790M,MET,TP53	Osimertinib	6

EGFR, epidermal growth factor receptor; TKI, tyrosine kinase inhibitor; ^*^censoring during the last follow-up. Survival time, Survival time after squamous cell transformation.

In the present case, the adenocarcinoma was confirmed to initially harbor the EGFR mutation, and responded well to icotinib. However, acquired resistance to icotinib was suspected due to the progressive disease. The pathological evidence revealed that the patient merely had squamous cell carcinoma. The accurate mechanism of the transformation from adenocarcinoma to squamous cell carcinoma remains unclear. The potential explanations for this observation are, as follows: (1) adenocarcinoma transformed to squamous cell carcinoma under the pressure of the EGFR-TKI treatment; (2) both types of cancers initially coexisted within the lesion (25), but only squamous cell carcinoma remained after treatment; (3) this was secondary to another primary cancer. In the present case, two key factors support the first potential mechanism: transformation to squamous cell carcinoma. One reason is that EGFR mutants are always detected in biopsies, and another reason is that the second sample was biopsied from the new lesion after icotinib treatment, and the lesion responded to osimertinib. The small biopsy size was a limitation in the present study. Hence, the samples could not well-represent the tumor. Furthermore, the squamous cell carcinoma may have become dominant during the disease progression. When the clinical response of the tumor was not initially expected, or when discordant responses are observed among different lesions in advanced lung adenocarcinoma, a repeat biopsy may be considered to determine the dominant histology that has not been initially detected. In addition, the role of the length of treatment with TKIs on the histological transformation remains unclear (24). Thus, further research is required to characterize these.

The transformation from adenocarcinoma to squamous cell carcinoma associated with the administration of EGFR-TKIs has been reported in a few cases. For example, the studies conducted by Yamaguchi F et al. (16) and Okabe N et al. (13) support the notion that third-generation EGFR TKIs, such as osimertinib, are effective for patients with squamous cell carcinoma transformations that harbor EGFR T790M mutations. In addition, Park et al. (26) and Chiang et al. (21) reported that partial remission was achieved after the administration of everolimus. In general, most patients do not receive effective treatment after EGFR-TKI resistance occurs. This hinders the development of a specific optimal treatment. Therefore, in order to help establish the best treatment for these patients, further studies are needed. In the present study, the patient was administered with osimertinib and crizotinib after the transformation, and transient remission was observed. However, the subsequent use of everolimus did not work. This may be explained by the tumor burden, and lack of PTEN and PIK3CA mutants. That is, the transient remission after the transformation demonstrates that chemotherapy and immunotherapy remain as effective tools for such cases.

In conclusion, the transformation of adenocarcinoma to squamous cell carcinoma associated with EGFR-TKI resistance is rare in clinical practice, and the number of reported cases remains few. The present report strengthens histological transformation as a source of acquired EGFR-TKI resistance. However, caution is required in practice for the possibility of pathological transformation. Furthermore, the accurate mechanism that involves the transformation remains unclear, and the optimal treatment for this situation should be evaluated in further studies.

## Data availability statement

The original contributions presented in the study are included in the article/supplementary material. Further inquiries can be directed to the corresponding author.

## Ethics statement

Ethical approval was not provided for this study on human participants because it is a case report and literature review. The patients/participants provided their written informed consent to participate in this study.

## Author contributions

Y-ZX and S-LZ designed/performed most of the investigation, data analysis and wrote the manuscript. LX provided pathological assistance. X-WT contributed to the interpretation of the data and analyses. All authors contributed to the article and approved the submitted version.

## Funding

This study was financially supported by the Health Commission of Hunan Province (No. 20201946).

## Conflict of interest

The authors declare that the research was conducted in the absence of any commercial or financial relationships that could be construed as a potential conflict of interest.

## Publisher’s note

All claims expressed in this article are solely those of the authors and do not necessarily represent those of their affiliated organizations, or those of the publisher, the editors and the reviewers. Any product that may be evaluated in this article, or claim that may be made by its manufacturer, is not guaranteed or endorsed by the publisher.
